# Reproductive Isolation between Host Races of *Phytomyza glabricola* on *Ilex coriacea* and *I. glabra*

**DOI:** 10.1371/journal.pone.0073976

**Published:** 2013-09-18

**Authors:** Julie B. Hebert, Sonja J. Scheffer, David J. Hawthorne

**Affiliations:** 1 Behavior, Ecology, Evolution, and Systematics Program, University of Maryland, College Park, Maryland, United States of America; 2 Department of Entomology, University of Maryland, College Park, Maryland, United States of America; 3 Systematic Entomology Laboratory, United States Department of Agriculture – Agricultural Research Service, Beltsville, Maryland, United States of America; Centro de Investigación y de Estudios Avanzados, Mexico

## Abstract

Recently diverged taxa often show discordance in genetic divergence among genomic loci, where some loci show strong divergence and others show none at all. Genetic studies alone cannot distinguish among the possible mechanisms but experimental studies on other aspects of divergence may provide guidance in the inference of causes of observed discordances. In this study, we used no-choice mating trials to test for the presence of reproductive isolation between host races of the leaf-mining fly, *Phytomyza glabricola* on its two holly host species, *Ilex coriacea* and *I. glabra.* These trials inform our effort to determine the cause of significant differences in the degree of divergence of nuclear and mitochondrial loci of flies collected from the two host plants. We present evidence of reproductive isolation between host races in a controlled greenhouse setting: significantly more mate pairs consisting of flies from the same host plant species produced offspring than inter-host mate pairs, which produced no offspring. We also tested whether the presence of the natal or non-natal host plant affects reproductive success. Flies collected from *I. coriacea* were more likely to produce offspring when in the presence of the natal host, whereas the presence or absence of either the natal or non-natal host had no effect on flies collected from *I. glabra*. The results indicate discordant patterns of nuclear and mitochondrial divergence among host races of *P. glabricola* are likely due to incomplete lineage sorting, and the host races may be well on their way to becoming biological species.

## Introduction

The evolution of new species results in genetic divergence among evolving lineages. The degree of genetic divergence among loci within the genome will vary depending on the differing effects of evolutionary mechanisms such as purifying selection (e.g., maintenance of housekeeping genes with reduced divergence [1] and divergent selection (e.g., genes involved in reproductive isolation with increased divergence [2]–[4]) across an organism’s genome(s). Unlike loci under selection, neutral regions are expected to accumulate differences primarily via the relatively slow mechanisms of mutation and genetic drift, resulting in much slower sorting of differences into alternative lineages [5], [6]. Neutral loci will also be more-affected by introgression among lineages, resulting in discordance among different areas of the genome, particularly between nuclear and organellar DNA (e.g., [7], [8]).

When there is discordance in the degree of divergence among different areas of the genome it can be difficult to distinguish introgression and episodic selection from incomplete lineage sorting as causes, particularly when relying on genetic data alone. One way to distinguish this pattern is to test for the presence or absence of reproductive isolation. When complete reproductive isolation exists between genetically divergent populations, introgression is unlikely and explanations for discordance between markers swing towards incomplete lineage sorting [8], and the populations may be biological species [9]. If currently diverged populations mate and produce offspring, then discordance among genomic regions may also be due to introgression of alleles (e.g. [7], [8]).

Populations seemingly in the midst of divergence and speciation [10], [11], such as host races [10]–[24], provide valuable case studies for the evolution of the genome during speciation. Host races imply genetically distinct populations that are associated with different hosts, such as in herbivorous insects [15], [18], [23], [25]–[30], parasites [31], [32], and parasitoids [33], [34]. Host races may have mosaics of high and low levels of genomic divergence resulting from divergent selection among hosts, [2] but whether or not undifferentiated regions are due to incomplete lineages sorting or introgression of neutral alleles is not always tested in these systems.

**Figure 1:**
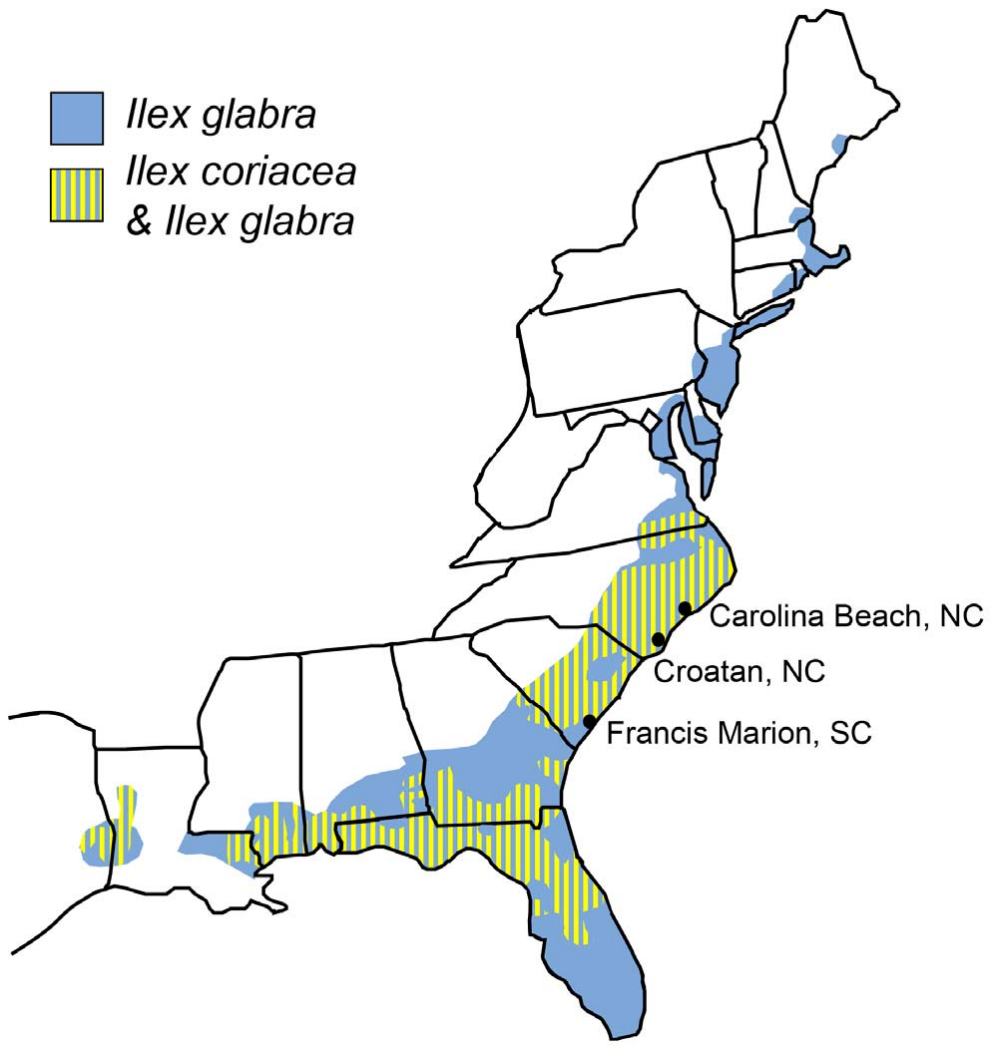
Endemic range of the host plants, *Ilex coriacea* and *I.* *glabra* with collection sites labeled.

Here, we address this most fundamental question of reproductive isolation using a newly studied host race system of a leaf-mining fly feeding on two species of holly, all of which are endemic to the eastern United States. *Phytomyza glabricola* Kulp belongs to a radiation of 14 closely related species, most of which are monophagous and all of which feed on hollies in the genus *Ilex* (Aquifoliaceae) [35]–[37]. Unlike most of its congeners, *P. glabricola* feeds on two sister species of holly, *Ilex glabra* (L.) A. Gray and *Ilex coriacea* (Pursh) Chapm. The distribution of *Ilex glabra* ranges from Maine to Florida and west to northeastern Texas (Figure 1). *Ilex coriacea*’s range is restricted to the southern portion of *I. glabra*’s range, where they are both sympatric and syntopic [38].

Adult flies that emerge from each host do not differ morphologically in either external characters or genitalia [37], [38]. However, when feeding on *I. coriacea, P. glabricola* (hereafter “coriacea-flies”) develop in approximately 9–10 months and are univoltine, whereas *P. glabricola* feeding on *I. glabra* (“glabra-flies”) develop in two to four weeks and are multivoltine [30], [35], [38], [39]. Despite these phenological differences, adult *P. glabricola* from both hosts emerge in synchrony in mid-January to mid-February [38], thus allowing the possibility of inter-host mating.

Initial work using amplified fragment length polymorphism (AFLP) frequencies revealed significant host-plant based genetic divergence in fly populations from North and South Carolina and host-associated clustering of flies in a neighbor-joining tree [30]. However, mitochondrial haplotypes did not cluster by host plant or location, and there were no fixed genetic differences between host races. One explanation for the observed discordance is that the populations are rapidly diverging in response to strong selection, causing the observed divergence of the AFLP frequencies, but the time has been insufficient for the mitochondrial haplotypes to have sorted into distinctive lineages using alternative host plants. Under such a scenario, we would expect flies reared on alternative host plant species not to produce viable hybrid offspring.

An alternative explanation is that the insects sampled from the alternative host plants are not accumulating genetic differences on their way towards speciation driven by host plant use, but rather the observed discordance between nuclear and mitochondrial markers are due to introgression of mitochondrial haplotypes (and neutral nuclear alleles) via an episodic response to differential selection by the two species of host plants. In this interpretation, the alternative host plants impose relatively strong selection on eggs, larvae, and/or pupae so that the individuals collected from each of those plants contain a subset of the genetic diversity in the full population. Adults from the sympatric natal plants mate and select host plants for oviposition randomly and this random mating homogenizes the genetic differences that accrue during pre-adult development. The random mating component of this scenario would lead us to expect flies from alternative host plant species to mate with each other and produce viable progeny at a rate similar to those reared from the same plant species.

Here, we evaluate these hypotheses by asking if laboratory crosses of holly leaf-miners reared from each of the two host plant species produce viable offspring at the same rate as do crosses of flies reared from the same host species. We used no-choice mating trials in a full factorial design of intra-host and inter-host mate pairs of flies to determine which fly combinations produced viable offspring. Our metric of number of viable offspring provides a measure of overall reproductive isolation that encompasses prezygotic (e.g., mating) as well as extrinsic and intrinsic postzygotic barriers (e.g., zygotic compatibility, oviposition, larval development, and successful emergence of adult flies).

In addition, we assessed the importance of the physical presence of the host plant species in mate choice and reproductive success. If flies prefer the natal host plant, we expected more successful trials when the natal host was present than when it was absent. In addition, we included trials containing both host plant species and recorded the species from which adult flies emerged to test whether the presence of the natal or non-natal host increased, or decreased, offspring production. Finally, we recorded the amount of time between the start of each trial and the emergence of offspring to identify the basis of differences in development time on each host plant species. In this study, female flies oviposited on both host plants and adult flies emerged from leaf-mines on both host plant species, but only for intra-host mate pairs, indicating the presence of reproductive isolation among host races of *P. glabricola*.

## Methods

### Collections

**Figure 2:**
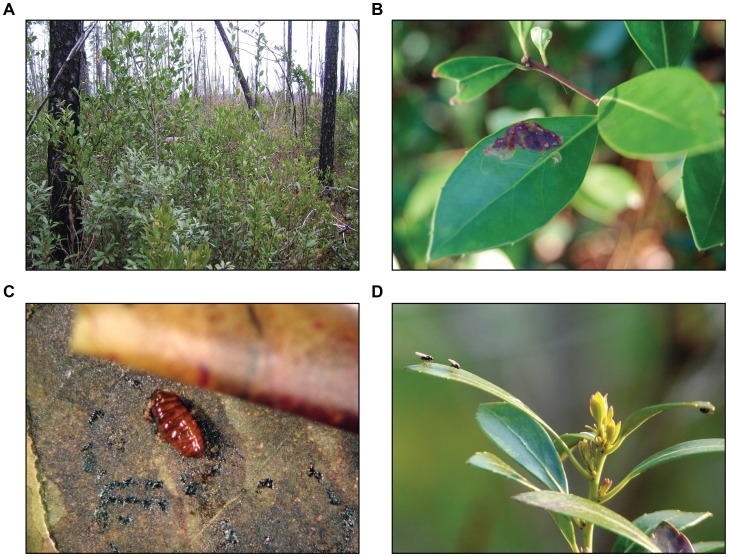
Images of *Phytomyza glabricola* and the native habitat. A. Typical habitat consisting of sympatric and syntopic *Ilex coriacea* and *I. glabra*. B. Leaf-mine on *I. coriacea*. C. Pupa revealed within leaf-mine on *I. glabra*. D. Adult *P. glabricola* on *I. glabra*.

Permits were obtained and plants collected from Carolina Beach State Park in North Carolina in September of 2005, and flies collected in January and February of 2006 from Croatan National Forest in North Carolina and Francis Marion National Forest in South Carolina (Figure 1). Leaves containing well-developed leaf-mines were removed from both host plant species at each site and brought back to the lab to rear pupae from the leaf-mines (Figure 2). Variation in leaf-mine abundance and rates of parasitism led to unequal sample sizes among locations. Pupae were dissected from mines, placed individually in 0.5 mL Eppendorf tubes, and stored in a moist chamber until the emergence of adults.

### Mating Trials

**Figure 3:**
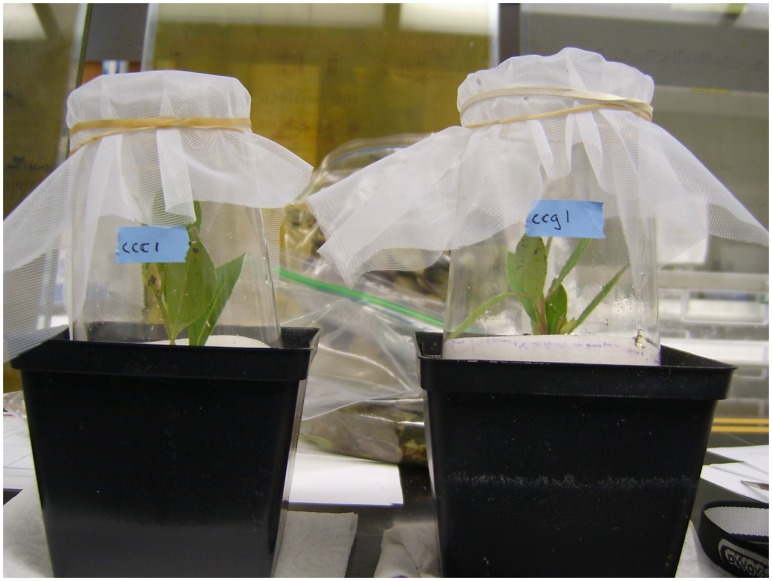
Mating chambers. A piece of foam surrounds the base of the plant in its pot, sealing the bottom portion of the cup. Fine mesh was held over the cup with a rubber band. Honey was placed on the side of the cup so that flies had a food source.

No-choice mating experiments were performed in modified 16 ounce plastic cups surrounding small propagated host plants in the greenhouse (Figure 3). A total of 107 trials were conducted using all combinations of male fly and female fly (from *I. coriacea* or *I. glabra*) placed in mating chambers with either *I. glabra*, *I. coriacea*, or both host plants present (Table 1). A small amount of honey was placed in each chamber as a potential food source for adult flies. As flies only live a few days in the greenhouse, pairs of flies were combined as soon as a male and female fly eclosed from the same location. The host plant(s) on which they were tested was randomized. Each trial was checked twice a day for the first 45 days, then weekly, to note formation of leaf-mines and the emergence of adults from pupae. Dead parental flies were removed from the cup, placed in 100% ethanol, and stored at −80°C. Oviposition punctures could not be identified under a dissecting scope, therefore oviposition could only be detected if eggs hatched and larvae formed a leaf-mine. In addition, despite numerous attempts to observe mating behavior, no matings were observed. Thus, prezygotic and postzygotic isolation could not be differentiated in this study. Trials either resulted in no leaf-mines or numerous leaf-mines, so to simplify analysis, a mating trial was considered successful if a mate pair produced offspring that eventually emerged as an adult from one of the leaf-mines inside the mating chamber, whereas trials were considered unsuccessful if no leaf-mine was formed after three months.

**Table 1:** Mating trials of *Phytomyza glabricola* on its host plants, *Ilex coriacea* and *I. glabra*.

Male fly		Female fly	Host-plant species present	# Successful Trials	Total # of Trials
Coriacea	×	Coriacea	*I. coriacea*	3	11
Coriacea	×	Coriacea	*I. glabra*	0	12
Coriacea	×	Coriacea	Both	4*	11
Coriacea	×	Glabra	*I. coriacea*	0	8
Coriacea	×	Glabra	*I. glabra*	0	8
Coriacea	×	Glabra	Both	0	8
Glabra	×	Coriacea	*I. coriacea*	0	5
Glabra	×	Coriacea	*I. glabra*	0	5
Glabra	×	Coriacea	Both	0	4
Glabra	×	Glabra	*I. coriacea*	2	12
Glabra	×	Glabra	*I. glabra*	3	12
Glabra	×	Glabra	Both	0	11
			Total	12	107

Trials were conducted in chambers containing either *I. coriacea* alone, *I. glabra* alone, or in the presence of both host plants. Trials were considered successful if the flies mated, the female oviposited eggs, and the offspring successfully emerged as adults.

* Offspring emerged from *I. coriacea* in two of the trials and from *I. glabra* in three of the trials.

Differences in the success rate between intra-host and inter-host mating trials were assessed using a Fisher’s exact test, allowing us to determine whether matings between different host races of *P. glabricola* were capable of producing adults that might allow for introgression of alleles among host races. First, the numbers of successful and unsuccessful trials were compared between intra-host and inter-host crosses to test for overall reproductive isolation. Next, the numbers of successful and unsuccessful trials were compared for the presence versus the absence of the natal host plant species to test whether the natal host species increased successful reproduction. Conversely, numbers of successful and unsuccessful trials were compared between the presence and absence of the non-natal host to test whether the non-natal host decreased reproductive success.

Flies lived an average of 2.6±0.08 days (mean ± SE, N = 279) after being placed in the mating chamber. Neither mating nor oviposition behavior was observed, but the amount of time in the chamber was relatively short, therefore development time was calculated for adult offspring by subtracting the start date of the trial from the date of emergence. Differences in development time among coriacea-flies and glabra-flies were assessed using a two-sample heteroscedastic t-test. All tests were performed in the statistical package R v2.7.2 [40].

## Results

Adult offspring were produced in 12 of the 107 trials, all of which were intra-host trials (Table 1). We observed a significantly greater success rate in intra-host trials (25% successful) than inter-host trials (0% successful) despite the low number of trials resulting in adult offspring (p = 0.0075). Because no inter-host trials produced offspring, the remaining results refer to intra-host trials only.

Natal host plant species presence increased the success of coriacea-flies (p = 0.0356), but had no effect on reproductive success of glabra-flies (p = 1; Table 2). On the other hand, the presence of the non-natal host did not affect the success rate of either host race (coriacea-flies: p = 0.5716; glabra-flies: p = 0.3126; Table 2). Interestingly, adult offspring emerged from both host plant species for both coriacea-fly and glabra-fly intra-host matings: offspring emerged from coriacea-fly intra-host trials on *I. coriacea* alone and from both *Ilex* species when both were present (Table 1). Offspring from glabra-fly intra-host trials emerged from trials on *I. coriacea* alone and from trials on *I. glabra* alone (Table 1).

**Table 2:** Effect of natal and non-natal hosts on successful reproduction in mating trials of *Phytomyza glabricola.*

		Natal		Non-natal	
Mate Pairs		Present	Absent	Present	Absent
Coriacea-Coriacea	Successful	7	0	4	3
	Unsuccessful	15	12	19	8
Glabra-Glabra	Successful	3	2	2	3
	Unsuccessful	20	10	21	9

The host plant species is considered present in trials containing only the host species in question and trials with both species present. Trials were considered successful if the flies mated, the female oviposited eggs, and the offspring successfully emerged as adults.

Development time in the greenhouse did not match expectations based on wild populations: all offspring that emerged from each host plant species did so within two months of the start of the trial. Offspring from coriacea-fly mate pairs emerged in 43±1.8 days (mean ± SE, N = 24) whereas offspring from glabra-fly intra-host mate pairs emerged in 52±6.4 days (mean ± SE, N = 5). The time between the start of a trial and the emergence of offspring did not significantly differ between coriacea-fly and glabra-fly intra-host crosses (t = 1.27, df = 4.63, p = 0.264).

## Discussion

A previous genetic study of host-associated populations of *P. glabricola* revealed conflicting results between nuclear DNA and mtDNA sequences; the former showed significant clustering of flies based on their host plant, whereas the latter showed no genetic structuring [30]. In this study, we used mating trials to determine whether the genetic discordance was due to incomplete lineage sorting, introgression, or an episodic response to divergent selection. We demonstrate the presence of reproductive isolation between host-associated populations of *P. glabricola* on its host plants, *I. coriacea* and *I. glabra*, suggesting the conflicting genetic patterns are likely due to incomplete lineage sorting.

### Reproductive Isolation

The lack of viable offspring from any inter-host mate pairs suggests the presence of prezygotic or postzygotic barriers to gene flow. These reproductive barriers indicate that genetic signatures of gene flow observed in a previous population-level study [30] are likely due to ancestral polymorphisms which remain shared because of incomplete lineage sorting.

### Host Plant Presence

Intra-host crosses were more successful than inter-host crosses regardless of the host species present. For glabra-flies reproductive success did not depend on which host plant species was present. However, coriacea-flies had increased reproductive success when *I. coriacea* was present, suggesting that coriacea-flies may obtain cues from *I. coriacea* that trigger mating or oviposition [41]–[47].

Offspring from all intra-host mate pairs emerged from both *I. coriacea* and *I. glabra*, regardless of the parents’ natal host, suggesting that host-mediated larval survivorship or development may not be driving the observed divergence.

### Development Time

Unexpectedly, all offspring of successful mate-pairs emerged in 31–65 days, irrespective of which host plant they, or their parents, emerged from. In the field, flies on *I. coriacea* typically take nine months to develop as opposed to two months for flies on *I. glabra* [38]. The shortening of the larval feeding time of *I. coriacea*-reared flies has been previously observed in a greenhouse in MN (Scheffer, unpublished data) indicating that the flies’ development time has at least some environmental component relating to the host plant. Currently, we do not know the causes of the reduction in development time of the experimental coriacea-flies, nor do we know whether this modification occurs in the field.

### Conclusions

Our results suggest that host-associated populations of *P. glabricola* are closer to the “species” end of the speciation continuum between populations and species. We found significant reproductive isolation among *P. glabricola* leaf-miners from the two holly species: no inter-host crosses resulted in offspring. Female flies oviposited on both host plant species, and the offspring survived on both the parental and non-natal hosts. Coriacea-flies had greater reproductive success in the presence of the natal host, whereas the presence or absence of either the natal or non-natal host had no effect on glabra-flies.

This work establishes that reproductive isolation exists between host races of *P. glabricola*, yet the lack of divergence of mitochondrial loci indicates that the lineages are young [30]. We can now begin to investigate the ecological, behavioral, and/or genetic factors causing barriers to gene flow in this system. Recently diverged lineages, such as these, are important resources for study of the process of divergence and speciation, allowing us the opportunity to determine the most important processes driving speciation, and further our understanding of how current biodiversity originally arose.
